# A cost-effective protocol for single-cell RNA sequencing of human skin

**DOI:** 10.3389/fimmu.2024.1393017

**Published:** 2024-10-30

**Authors:** Saba Khoshbakht, Özgür Albayrak, Ergün Tiryaki, Orhan Ağcaoğlu, Ayşe Öktem, Gizem Pınar Sun, Elif Er Gülbezer, Sümeyre Seda Ertekin, Ayşe Boyvat, Atay Vural, Seçil Vural

**Affiliations:** ^1^ Graduate School of Health Sciences, Koç University, Istanbul, Türkiye; ^2^ Koç University Research Center for Translational Medicine, Koç University, Istanbul, Türkiye; ^3^ Department of Surgery, Koç University School of Medicine, Istanbul, Türkiye; ^4^ Department of Dermatology, Ankara University Faculty of Medicine, Ankara, Türkiye; ^5^ Department of Dermatology, Başakşehir Çam ve Sakura Şehir Hastanesi, Istanbul, Türkiye; ^6^ Department of Rheumatology, Koç University School of Medicine, Istanbul, Türkiye; ^7^ Department of Dermatology, Koç University School of Medicine, Istanbul, Türkiye; ^8^ Department of Neurology, Koç University School of Medicine, Istanbul, Türkiye

**Keywords:** skin, inflammation, single cell RNA sequencing (scRNA), flow cytometry, souporcell, multiplexing, skin dissociation

## Abstract

**Introduction:**

Single-cell RNA sequencing (scRNAseq) and flow cytometry studies in skin are methodologically complex and costly, limiting their accessibility to researchers worldwide. Ideally, RNA and protein-based analyses should be performed on the same lesion to obtain more comprehensive data. However, current protocols generally focus on either scRNAseq or flow cytometry of healthy skin.

**Methods:**

We present a novel label-free sample multiplexing strategy, building on the souporcell algorithm, which enables scRNAseq analysis of paired blood and skin samples. Additionally, we provide detailed instructions for simultaneous flow cytometry analysis from the same sample, with necessary adaptations for both healthy and inflamed skin specimens.

**Results:**

This tissue multiplexing strategy mitigates technical batch effects and reduces costs by 2-4 times compared to existing protocols. We also demonstrate the effects of varying enzymatic incubation durations (1, 3, and 16 hours, with and without enzyme P) on flow cytometry outcomes. Comprehensive explanations of bioinformatic demultiplexing steps and a detailed step-by-step protocol of the entire experimental procedure are included.

**Discussion:**

The protocol outlined in this article will make scRNAseq and flow cytometry analysis of skin samples more accessible to researchers, especially those new to these techniques.

## Introduction

Analyzing RNA and protein at the single-cell level in lesional skin is crucial for understanding the immune mechanisms underlying dermatological disorders and identifying novel therapeutic targets. Recent advancements in standardized tissue dissociations and single-cell RNA sequencing (scRNAseq) systems have facilitated studies on diseases such as psoriasis and atopic dermatitis using single-cell analysis methods (1–4). These studies have significantly enhanced our understanding of the immune mechanisms involved in these disorders. However, the complex methodology and high costs associated with these studies limit their accessibility, preventing many laboratories from conducting such experiments and hindering the application of these studies to other dermatological disorders. Therefore, there is a pressing need for cost-effective and detailed experimental protocols to make these techniques more accessible to researchers.

Obtaining high-quality cells with intact RNA and protein epitopes from solid tissues has been a significant challenge for single-cell studies. Numerous protocols for tissue dissociation have been documented in the scientific literature (5–7). The introduction of automated tissue dissociator systems has further facilitated flow cytometry and scRNAseq analysis of dissociated skin cells (8, 9). Recently, comprehensive methodological papers have provided efficient and optimized protocols for scRNAseq and flow cytometry analysis of human and pig skin, which we recommend for further reading (5, 10–12). However, these papers predominantly focus on either scRNAseq or flow cytometry, without testing both methods on the same sample, and primarily concentrate on healthy skin. Inflamed skin, which typically has a higher cell count than healthy skin, may require different experimental conditions.

High-throughput single-cell multi-omics methods, such as CITE-Seq, have recently been developed to simultaneously study RNA and protein. However, these techniques are less sensitive to dim cell surface markers. Conventional and spectral flow cytometry, as well as mass cytometry, remain the gold standards for single-cell protein-based studies. Preservation of cell surface epitopes is essential for these methodologies and can be influenced by enzyme selection and prolonged enzymatic incubation periods. Studies systematically comparing the effects of enzymatic incubation durations are scarce, and none have examined the impact of enzyme P, commonly used in cell dissociation protocols to increase cell yield in scRNAseq studies.

Sample multiplexing is vital for reducing technical batch effects and experimental costs in scRNAseq studies. Traditional sample multiplexing relies on oligonucleotide-conjugated hashtag antibodies. However, this approach has limitations, including the risk of inefficient antibody binding to all cells, additional material requirements for both multiplexing and demultiplexing steps, and increased experimental complexity and costs. Existing protocols often lack detailed steps for multiplexing samples, library generation for barcodes, and demultiplexing methods. In our experience, initial attempts at sample multiplexing in our laboratory faced significant challenges due to insufficient detail in existing protocols, risking the loss of samples and materials, which prompted us to look for alternative sample multiplexing and demultiplexing methods.

Recently, algorithms that recognize individual-specific single nucleotide polymorphism signatures have been developed for label-free demultiplexing of pooled samples. One such method, souporcell, has shown excellent benchmarking results against antibody-based cell hashing techniques (13). However, the complex experimental design proposed in the original article, which involves splitting the same sample into multiple experimental batches, has limited its usage in research projects. A novel experimental design is needed to make this method more widely applicable.

In this study, we aimed to establish a practical and cost-effective protocol for single-cell protein and RNA analysis. We provide tailored instructions for both healthy and lesional skin, demonstrating the influence of various enzymatic incubation conditions on flow cytometry analysis. Importantly, we introduce a novel, two-layered sample multiplexing design that combines two complementary strategies from the literature, along with demultiplexing steps, for scRNAseq experiments. This approach can lower the experimental costs 2-4 fold. Detailed explanations for each optimization step are provided, allowing researchers to adapt and customize protocols according to their specific needs (**Supplementary Material**).

## Materials and methods

### Experimental design

**Figure 1:**
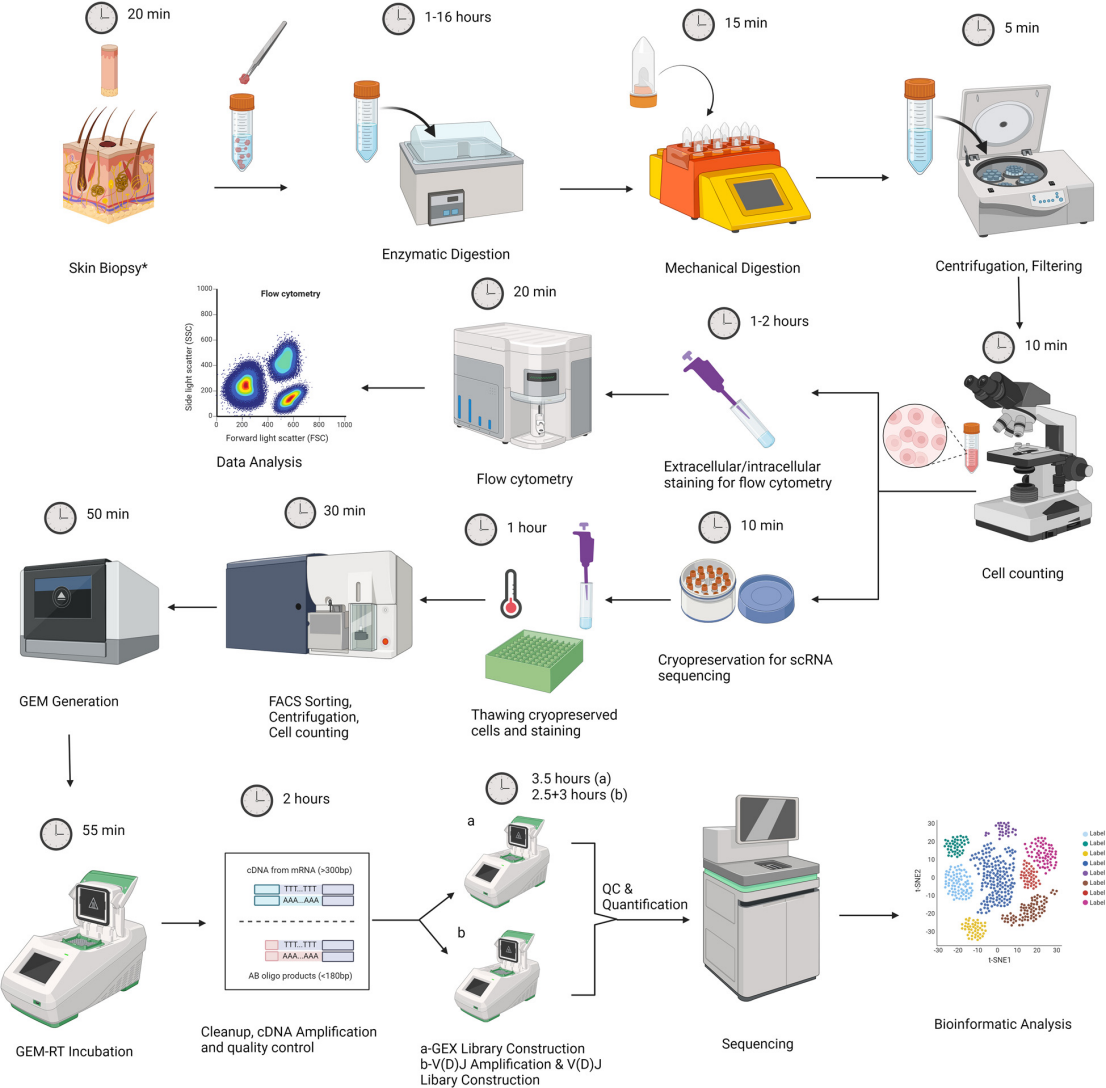
Graphic representation of our experimental workflow that involves skin biopsy, tissue dissociation using the Whole Skin Dissociation Kit (Miltenyi Biotec, Germany) in 2 steps of enzymatic and mechanical dissociation, staining part of the dissociated cells for flow cytometry analysis, and cryopreservation part of cells for subsequent scRNAseq analysis. Second part includes thawing of cell suspensions, sample preparation by Fluorescence-activated cell sorting (FACS), cell counting by trypan blue exclusion method, Gel Beads-in-emulsion (GEM) generation, library preparation, sequencing and data analysis. The figure was created with BioRender.com. *For lesional skin, one 4mm punch biopsy was obtained. For healthy skin samples, two 6 mm specimens were obtained.

Our experimental design consists of two main parts. The first part includes the acquisition of skin samples, followed by tissue dissociation and flow cytometry analysis of freshly isolated cells. A fraction of dissociated skin cells are cryopreserved for future scRNAseq analysis at the end of this step (**Figure 1**). The second part begins with sample preparation for scRNAseq analysis by cell sorting, followed by gel beads-in-emulsion (GEM) generation, library construction, sequencing, and data analysis (**Figure 1**). A step-by-step protocol is presented in the **Supplementary Materials**.

### Sample collection

The study was approved by the Koç University Committee on Human Research (protocol number 2022.058.IRB2.007). Informed consent was obtained from all participants.

Healthy skin samples were obtained by surgical excision of excess skin tissue from individuals undergoing surgery (n=6). Tissues were kept in cold tissue storage solution (130-100-008, Miltenyi Biotec, Germany) until delivered to the laboratory on ice. From each large surgical excision, eight 6 mm punch biopsies were obtained. These punch biopsies were then distributed as two pieces for each experimental condition, including three different enzyme incubation periods and treatment with enzyme P. Lesional skin biopsy specimens were collected from patients with Behçet’s disease (BD, n=12). A 4 mm punch was used to collect skin biopsy samples from the lesional area. Demographic and clinical characteristics of the study samples are demonstrated in **Table 1**.

**Table 1:** Demographic and clinical characteristics of the study samples, including Behcet’s disease (BD) patients and healthy controls.

Characteristic	BD (n=12)	HC (n=6)
Age: median (Interquartile Range)	35 (30-41)	70 (65-73)
Gender (Male: Female)	6:6	0:6
Smoking	3/12	1/6
HLA-B51 positivity	4/9	–
Lesion Type:		
Erythema Nodosum-like lesions (EN)	3	–
Papulopustular Eruptions (PPE)	6	–
Genital Ulcers (GU)	3	–

Data for age are presented as median with interquartile range (IQR). Gender is reported as a ratio of male to female participants. Smoking status and HLA-B51 positivity are also included. For patients, lesion types are categorized as erythema nodosum-like lesions (EN), papulopustular eruptions (PPE), and genital ulcers (GU).

For the isolation of peripheral blood mononuclear cells (PBMCs), venous blood was diluted 1:1 with phosphate-buffered saline (PBS) and spread over an equal volume of Lymphoprep 1.077g/ml density gradient (Axis-Shield, Norway) in 50 ml Falcon tubes. The tubes were centrifuged at 500g for 30 minutes at room temperature without brakes. Following centrifugation, the PBMC layer was transferred into another 50 ml sterile tube and washed with PBS containing an equal volume of 1% BSA. A fraction of cells were analyzed freshly by flow cytometry and the remaining cells were frozen in the cryopreservation solution (10% DMSO, 90% FBS) for later usage.

### Skin dissociation

After the delivery of skin samples, the tissue was washed thoroughly with PBS, and subcutaneous tissue was removed with a scalpel, paying attention to keeping the dermis intact. Then, the skin tissues were dissociated enzymatically and mechanically using the Whole Skin Dissociation Kit (130-101-540, Miltenyi Biotec, Germany) and gentleMACS Octo Dissociator with Heaters (Miltenyi Biotec, Germany) following the manufacturer’s protocol. The kit recommends the use of three enzymes (enzymes A, D, and P) for enzymatic dissociation, with the usage of enzyme P being optional depending on the subsequent analysis method. Enzyme P is known to cause cleavage of some extracellular epitopes, which may interfere with flow cytometry analysis. The duration of enzymatic incubation can be either 3 hours or overnight as per the manufacturer’s manual. To investigate the effect of enzyme P and various incubation durations (1h, 3h, and 16h) on flow cytometry results, we first conducted a systematic study, as these factors have not been studied comprehensively before.

The enzymatic steps were carried out as follows: 435 µL of Buffer L and, if applicable, 12.5 µL of Enzyme P were combined in a gentleMACS C tube. Subsequently, 50 µL of Enzyme D and 2.5 µL of Enzyme A were added to the mixture, which was then thoroughly mixed. The tissue-enzyme mixture was then placed in a 37˚C water bath for incubation. We compared three different incubation durations: 1, 3, and 16 hours.

After incubation, 500µL of cold DMEM (Gibco, USA) was added to the mixture, and tubes were placed onto the gentleMACS system. The “h_skin_01” program was initiated to mechanically dissociate the tissues into cell suspension. Following completion of the program, samples were briefly centrifuged and filtered using a 70 µm cell strainer (83.3945.070, Sarstedt, Germany), with 4 ml of DMEM used to wash the cells.

After centrifugation at 350g for 10 minutes, the cell pellet was counted using a hemocytometer with 0.4% Trypan blue to exclude dead cells. Approximately 10^5^ cells (in 100 µL buffer) were stained and analyzed by flow cytometry on the same day, while the remaining cells were cryopreserved (in 1 mL fetal bovine serum plus 10% DMSO) for subsequent scRNAseq analysis.

### Flow cytometry

All antibodies and the fixable viability dye used in this study are listed in **Supplementary Table 1**. Initially, 10^5^ freshly isolated skin cells or PBMCs were incubated with Zombie NIR fixable viability dye for 10 minutes on ice. Subsequently, 2 ml of FACS buffer (PBS+ 1% BSA) was added to the tubes, followed by centrifugation at 500 g for 5 minutes. After centrifugation, the supernatant was decanted, and a cell surface antibody cocktail was added. Samples were then incubated for 20 minutes on ice. Post-incubation, samples were washed again with 2 ml of FACS buffer and the supernatant was removed. For intracellular staining, cells were fixed with 500 µL of Fixation Buffer (420801, BioLegend, USA) for 20 minutes at room temperature and directly centrifuged at 500 g for 5 minutes. Following the removal of the supernatant, cells were washed with Intracellular Staining Permeabilization Wash Buffer (421002, BioLegend, USA) and incubated with an intracellular antibody cocktail for 20 minutes at room temperature. After the incubation period, cells were washed with the Permeabilization Wash Buffer and the pellet was resuspended with 500 µL of FACS buffer. The acquisition was performed using a CytoFLEX SRT (Beckman Coulter) flow cytometer, and the results were analyzed using FlowJo v.10.9.0 (BD Biosciences, USA). The markers used to identify the cell populations of interest are listed in **Supplementary Table 2**.

### Sample preparation for single-cell RNA sequencing

For successful single-cell RNA sequencing using the 10X Chromium system, proper sample preparation is paramount. The viability and concentration of cells for GEM generation must adhere to the manufacturer’s protocol. A live cell ratio exceeding 90% is highly advisable, although ratios above 60-70% can also be attempted with potentially reduced success rates.

In this study, we adopted a sample pooling strategy to mitigate batch effects and lower experimental costs by combining two or more samples. Initially, cryopreserved cell suspensions from skin and peripheral blood were thawed. Subsequently, cells were stained with fixable viability dye (Zombie NIR) in PBS, followed by anti-human CD45 PE-Cy5 in FACS buffer (PBS+ 1% BSA). CD45+ live cells were then simultaneously sorted from two samples using the CytoFLEX SRT (Beckman Coulter) and FACS Aria III cell sorters (BD Biosciences, USA). During sorting, a 100 µm nozzle was utilized, with a constant pressure of 20 psi, and the sample chamber was maintained at 4°C. The sorting speed was kept low (1500 events/seconds) to ensure high viability and purity.

The manufacturer’s protocol for the Chromium Next GEM Single Cell 5’ Reagent Kit V2 (Dual Index, 10X Genomics, USA) used for this study suggests an ideal cell concentration of 700 to 1200 cells per microliter for GEM generation. However, cell sorting from skin tissue often results in concentrations lower than this range. In such cases, it is necessary to concentrate the cell suspension after sorting. In the current study, this was accomplished by an additional centrifugation step at 850 g for 5 minutes. After carefully removing the supernatant, the cells were reconstituted in the desired volume.

### Library preparation and sequencing

Once the sample preparation step was completed by reaching the optimum cell concentration, steps for cDNA preparation, amplification, and library preparation were performed by using the Chromium Controller and the Chromium Next GEM Single Cell 5’ Reagent Kits V2 (Dual Index, 10X Genomics, USA).

First, GEMs were generated by loading a master mix containing cells, gel beads, and partitioning oil on the Chromium Next GEM Chip K and running the Chromium Controller system. Next, gel beads were dissolved, cells were lysed and 10X barcoded cDNAs were produced from poly-adenylated mRNAs. The barcoded cDNAs were purified from the reaction mixture using Silane magnetic beads and amplified via PCR. The quality control (QC) and quantification were performed by Agilent 2100 Bioanalyzer (Agilent Technologies, USA). For Gene Expression (GEX) library construction enzymatic fragmentation, size selection was performed to get the optimal cDNA amplicon lengths. Finally, sequencing-ready dual index libraries were prepared by end repair, A-tailing, adaptor ligation, and sample index PCR.

Paired-end sequencing was performed on the Illumina NovaSeq 6000 platform by outsourcing to a service provider. A minimum reading depth of 20,000 reads per cell was utilized.

### Alignment and demultiplexing of single-cell RNAseq data

The raw FASTQ files were aligned to the reference genome (GRCh38) using Cell Ranger (v.7.1.0) multi pipeline. Both reference genome and Cell Ranger software were obtained from the official 10X Genomics website.

We used souporcell for demultiplexing of pooled samples (13). The BAM file generated by Cell Ranger was plugged into the souporcell pipeline. This method utilizes single nucleotide polymorphisms (SNPs) detected in scRNA-seq reads to demultiplex scRNA-seq data originating from different individuals (13). This *in silico* method is freely available and label-free. The souporcell pipeline was executed on a high-performance computing cluster using the singularity image provided by souporcell authors. The analysis employed the “*souporcell_pipeline.py*” script and utilized the same reference transcriptome used during alignment.

### Identification of paired samples and donor sex in dual reactions

After demultiplexing of pooled samples by souporcell, the identity of each cluster was determined based on: 1) The presence of matched skin and PBMC pairs, which have the same genotype in dual reactions, and 2) the Identification of the donor’s sex. Skin and PBMC samples from the same donor were identified using the “*shared_samples.py*” module of the souporcell. The donor’s sex for each souporcell cluster was determined by using a subset of Y chromosome genes including ZFY, RPS4Y1, EIF1AY, KDM5D, NLGN4Y, TMSB4Y, UTY, DDX3Y, and USP9Y (14).

### Analysis of single-cell RNAseq data

After alignment and demultiplexing, count matrices were processed using Scanpy. Cells with less than 200 genes and genes expressed in fewer than 3 cells were filtered. Moreover, cells containing more than 4000 genes were excluded. In the doublet detection part, cross-genotype doublets, as detected by souporcell, were eliminated, and Scrublet was employed to identify doublets based on expression profiles.

Subsequently, the data was normalized to 10,000, and the log(x+1) transformation was applied. Feature selection was performed using the “sc.pp.highly_variable_genes” function, and principal component analysis (PCA) was computed on the scaled expression matrix of the highly variable genes. BBKNN’s ridge regression function was utilized to eliminate technical confounders such as donor-specific variation and count depth, while preserving biological variation such as cell types and disease effects (15, 16). Following this preprocessing step, different pools were integrated using the Harmony algorithm (17). A neighborhood graph and UMAP embedding were computed based on the harmony-corrected principal components.

Cell type annotation was done by CellTypist using pre-trained built-in models such as “Immune_All_High.pkl” and “Immune_All_Low.pkl” (18). During cell type prediction, the majority voting classifier is enabled to increase the accuracy of annotation.

### Statistical analysis

Repeated Measures One-Way ANOVA was used to compare different incubation durations. Paired T-test was used to assess the effect of enzyme P. Statistical analysis was conducted by GraphPad Prism v8 (GraphPad Software, USA).

## Results

### Identification of immune cell subsets and intracellular cytotoxic molecules in skin by flow cytometry

We initially dissociated healthy skin samples by incubating them with enzymes A and D (without enzyme P) for three hours and analyzed them freshly after staining with flow cytometry. Our gating strategy and fluorescence-minus-one (FMO) controls used for dimly expressed markers are illustrated in **Supplementary Figures 1, 2**, respectively. Using the antibody panel in this study, we could readily identify T cell subsets, including CD4+ T helper cells, CD8+ cytotoxic T cells, CD69+ resident memory T cells, as well as natural killer (NK) cells. Additionally, we were able to detect intracellular granzyme B and granzyme K expression in cytotoxic T cells.

### The impact of enzymatic incubation duration and enzyme P on cell counts and cell type frequencies

**Figure 2:**
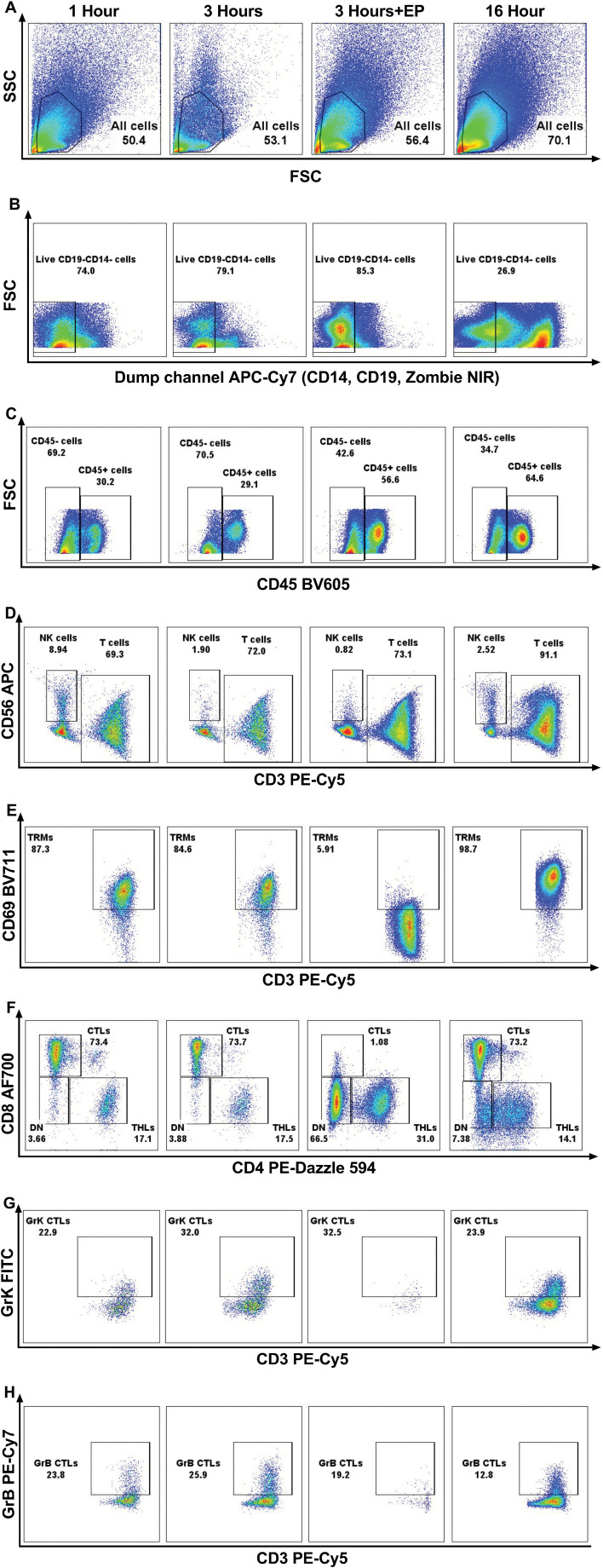
Representative flow cytometry graphs demonstrating the effect of enzymatic treatment for 1 hour, 3 hours, 16 hours; and 3 hours of incubation using enzyme P on all cells in healthy skin **(A)**, live CD19-CD14- cells **(B)**, CD45+ and CD45- cells **(C)**, CD3+ T cells and CD56+ NK cells **(D)**, CD69+ resident memory T cells (TRMs) **(E)** CD4+ T helper cells (THLs) and CD8+ cytotoxic T cells (CTLs) **(F)**, Granzyme K+ (GrK) CTLs **(G)**, and Granzyme B+ (GrB) CTLs **(H)**. It is demonstrated that, due to the cleavage of CD69 and CD8 antigens by enzyme P, the population of TRMs **(E)** and CTLs **(F)** decreased dramatically.

**Figure 3:**
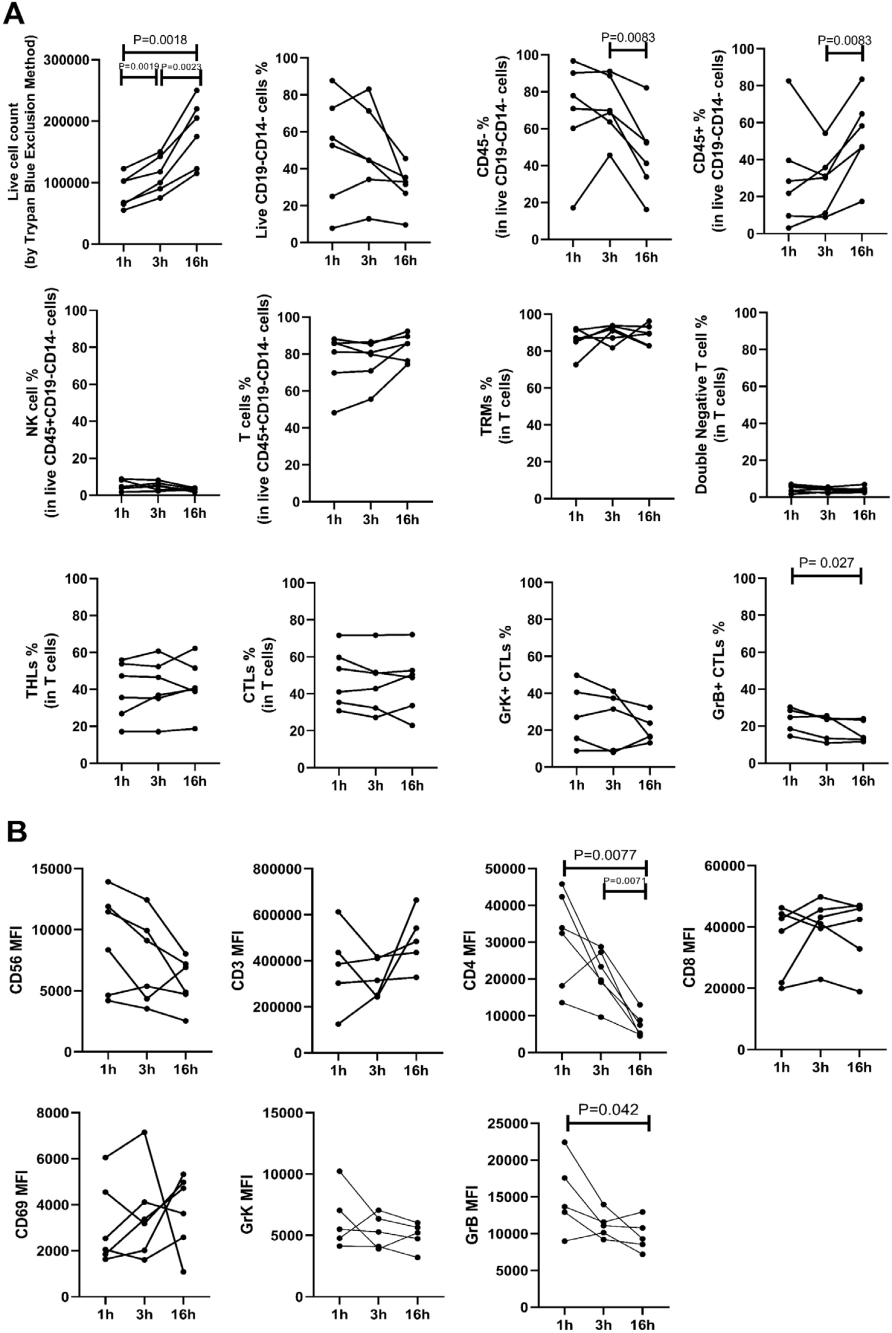
The impact of various enzymatic incubation periods (1 hour, 3 hours, 16 hours) on cell percentages **(A)** and mean fluorescence intensity (MFI) measurements **(B)** is shown. A) Live cell counts (determined by trypan blue exclusion method) increased with longer incubation times, indicating enhanced cell yield. The percentage of viable CD14-CD19- leukocytes, assessed by flow cytometry, showed no significant difference across the three incubation periods. However, the percentage of CD45- cells decreased, leading to a significant increase in CD45+ percentage after 16 hours compared to 3 hours of incubation. There was no notable difference in the percentage of lymphocyte subtypes across different incubation times, except for a decrease in CD8+Granzyme B+ cytotoxic T lymphocytes (CTLs) after 16 hours compared to 1 hour incubation. B) MFI values of CD3, CD8, CD56, CD69, and Granzyme K (GrK) showed no significant difference following enzymatic incubation for different durations. However, CD4 and Granzyme B (GrB) MFI decreased significantly after 16 hours of incubation.

The impact of varying incubation periods and the presence of enzyme P on selected extracellular and intracellular markers is illustrated in **Figure 2**. We observed a significant increase in the yield of trypan blue-negative live cells with longer incubation durations (**Figure 3A**). Specifically, samples subjected to a 1-hour incubation displayed a live cell count ranging from 5.5 × 10^4^ to 1.22 × 10^5^ per one 6 mm punch specimen, which was deemed suboptimal for subsequent applications. In contrast, samples incubated for 3 hours yielded a live cell count ranging from 7.5 × 10^4^ to 1.5 × 10^5^ per one 6 mm punch specimen, while a 16-hour incubation resulted in a live cell count ranging from 1.15 × 10^5^ to 2.5 × 10^5^ per one 6 mm punch specimen.

The percentage of CD45+ leukocytes was higher after 16 hours of incubation compared to 3 hours. The frequency of T cells, NK cells, CD4+ T helper lymphocytes (THL), CD8+ cytotoxic T lymphocytes (CTL), double negative cells, and CD69+ resident memory T (TRM) cells remained similar between groups (**Figure 3A**). However, there was a decline in the percentage of granzyme B-positive cytotoxic T lymphocytes (CTLs) after 16 hours of incubation compared to 1 hour (median, range: 24.8, 14.5-30.3 vs 13.8, 11.6-24.0; p=0.027), while the ratio of granzyme K-expressing CTLs remained unchanged. The mean fluorescent intensity (MFI) of CD3, CD8, CD56, CD69, and granzyme K was similar between groups. However, there was a significant decrease in the MFI of CD4 with longer enzymatic incubation periods (1h vs 16h: 33169, 13566-45794 vs 6387, 4494-12979; p=0.0077, 3h vs 16h: 21511, 9628-28753 vs 6387, 4494-12979; p=0.0071), and a decline was seen in Granzyme B MFI after 16 hours of incubation (1h vs 16h, p=0.042) (**Figure 3B**).

**Figure 4:**
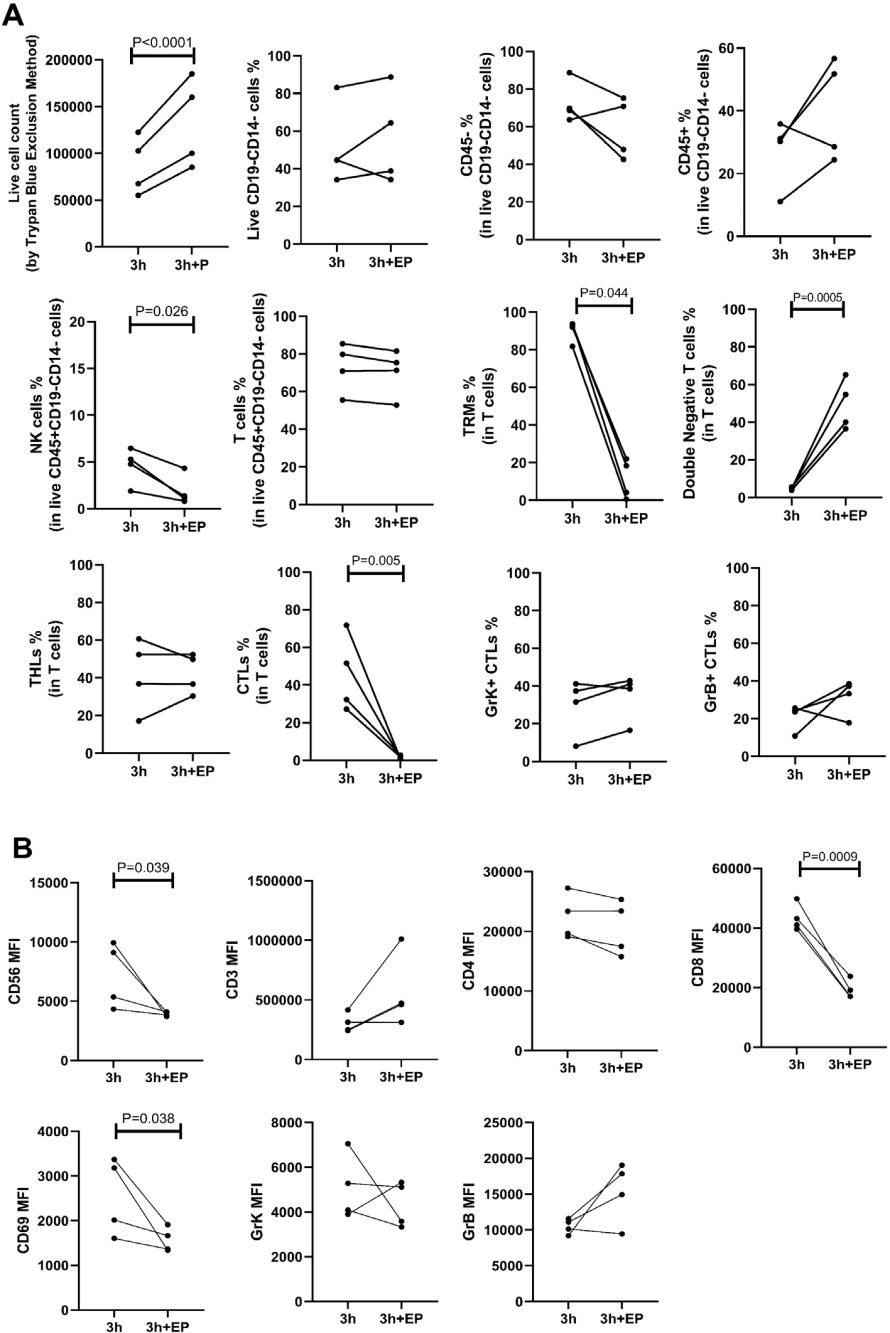
The impact of enzyme P on all cell percentages **(A)** and mean fluorescence intensity (MFI) measurements **(B)** is shown. A) Live cell counts demonstrated increased cell yield with the use of enzyme P. However, the percentage of NK cells, CD69+ cells, and CD8+ cytotoxic T lymphocytes (CTLs) significantly declined when enzyme P was used. B) The MFI of CD8, CD56, and CD69 significantly decreased when enzyme P was used in the enzymatic incubation. Statistical analyses were performed using Repeated Measures ANOVA (to compare different time points) and paired ratio T-test (for comparison of the effect of enzyme P).

We investigated the impact of enzyme P during a 3-hour incubation period. The live cell count per one 6 mm punch specimen after 3 hours of incubation without enzyme P ranged from 7.5 × 10^4^ to 1.5 × 10^5^, while with the addition of enzyme P, it increased significantly to 8.5 × 10^4^ to 1.85 × 10^5^ (p<0.0001) (**Figure 4A**). The use of enzyme P did not significantly alter the percentages of T cells, CD4+ T cells, granzyme K positive CTLs, or granzyme B positive CTLs. However, the percentages of CD8+ CTLs (41.95, 27.2-71.7 vs 1.44, 0.83-2.67; p=0.005) and CD69+ TRM cells (92.7, 81.8-93.8 vs 11.27, 0.52-22; p=0.044) decreased dramatically. Additionally, there was a statistically significant reduction in the percentage of NK cells after incubation with enzyme P (5.03, 1.90-6.48 vs 1.25, 0.82-4.33; p=0.026) (**Figure 4A**). Moreover, as anticipated, there was a significant decrease in the MFI of CD8 (42108, 39568-49811 vs 18090, 16887-23848; p=0.0009), CD56 (7239, 4347-9927 vs 3964, 3716-4110; p=0.039), and CD69 (2598, 1606-3370 vs 1515, 1339-1911; p=0.038) in the group treated with enzyme P compared to the group without enzyme P (**Figure 4B**).

Based on the results of these experiments, we decided to use a 3-hour enzymatic incubation without enzyme P for the analysis of lesional skin for simultaneous flow cytometry and scRNAseq analysis.

### Determination of tissue size required for flow cytometry and scRNAseq analysis in the inflamed skin

In prior methodological studies, the necessary size of skin tissue for scRNAseq analysis was determined. Given the higher concentration of inflammatory cells in inflamed skin, we hypothesized that a smaller tissue sample would be adequate for subsequent analysis compared to healthy skin.

For healthy skin specimens, a single 6 mm punch biopsy provided an average cell count of 1.125 × 10^5^ after a three-hour enzymatic incubation without enzyme P (range: 0.75 × 10^5^ to 1.5 × 10^5^, n=6). This quantity is sufficient for flow cytometry analysis of lymphocytes. However, if scRNAseq is also intended, we recommend using a second biopsy specimen.

Conversely, we discovered that after three hours of enzyme incubation without enzyme P, inflamed skin tissue measuring 4 mm in diameter yielded an average of 4.6 ×10^5^ live cells (range: 8 × 10^4^ - 9.7 × 10^5^, n=12). Despite some variability between samples, this amount is typically adequate for both flow cytometry and scRNAseq analysis.

### The effect of cryopreservation on dissociated skin cells

Sample multiplexing can help avoid technical batch effects and reduce the cost of single-cell RNA sequencing (scRNAseq). However, multiplexing freshly isolated cells can be challenging due to the need for synchronized sample collection from different individuals. Cryopreservation of isolated cell suspensions offers a solution to this issue. In our study, we stored dissociated skin cells in liquid nitrogen for subsequent cell sorting and scRNAseq analyses. This approach allowed us to compare the viability of CD45+ cells in freshly isolated and thawed samples. We found that the live CD45+ cell percentage was consistently above 85% in both freshly isolated and thawed skin cells, with no statistically significant difference between the two groups (**Supplementary Figure 3**).

### Sample multiplexing strategy for batch scRNAseq analysis of paired blood and skin samples

Current sample multiplexing method for scRNAseq analysis rely on the usage of sample barcoding kits. In this study, our aim was to conduct single-cell RNA sequencing (scRNAseq) analysis of matched skin and peripheral blood samples from various subjects in batches. To achieve this, we developed a novel sample multiplexing strategy that is based on a recently published label-free demultiplexing algorithm called “souporcell,” which utilizes distinct single nucleotide polymorphism (SNP) patterns unique to genetically different individuals (13). Our strategy represents a modified and enhanced version of the multiplexing strategy described in the original paper and allows us to multiplex two or more samples without using any additional tissue barcoding steps.

In the first strategy, two samples can be multiplexed using a relatively simple design, similar to the approach outlined in the original article (13). To implement this strategy, the PBMC and dissociated skin samples from the same subject are placed in two separate tubes. Additionally, one genetically unrelated sample is added to each tube (these additional samples should not be paired samples) (**Table 2**). Subsequently, scRNAseq analysis of these four samples is conducted in two separate reactions. The demultiplexing of samples in each reaction is performed with souporcell, where the paired samples in different tubes belonging to the same individual can be readily identified based on their common SNP pattern. As the identity of paired samples is known beforehand, these clusters can be annotated with their respective sample identities. Once one of the sample identities is revealed, it becomes straightforward to identify the remaining sample in each tube. Using this strategy, it is possible to process nine pairs of PBMC and skin samples in 9 reactions, instead of 18, effectively reducing the number of necessary reactions by half.

**Table 2:** Multiplexing strategy for scRNAseq analysis 18 paired skin and peripheral blood samples obtained from nine different individuals.

Tube/ Reaction no:	Tissue:	Subject code:								
		S1	S2	S3	S4	S5	S6	S7	S8	S9
I	Skin	X	X							
II	PBMC		X	X						
III	Skin				X	X				
IV	PBMC					X	X			
V	Skin							X	X	
VI	PBMC								X	X
VII	Skin			X			X			
VIII	PBMC	X			X					
IX	Skin + PBMC							X (PBMC)		X (Skin)

The PBMC and dissociated skin sample pairs from the same subject were placed separately in two tubes/reactions. Then, one genetically unrelated sample was added to each tube.

**Figure 5:**
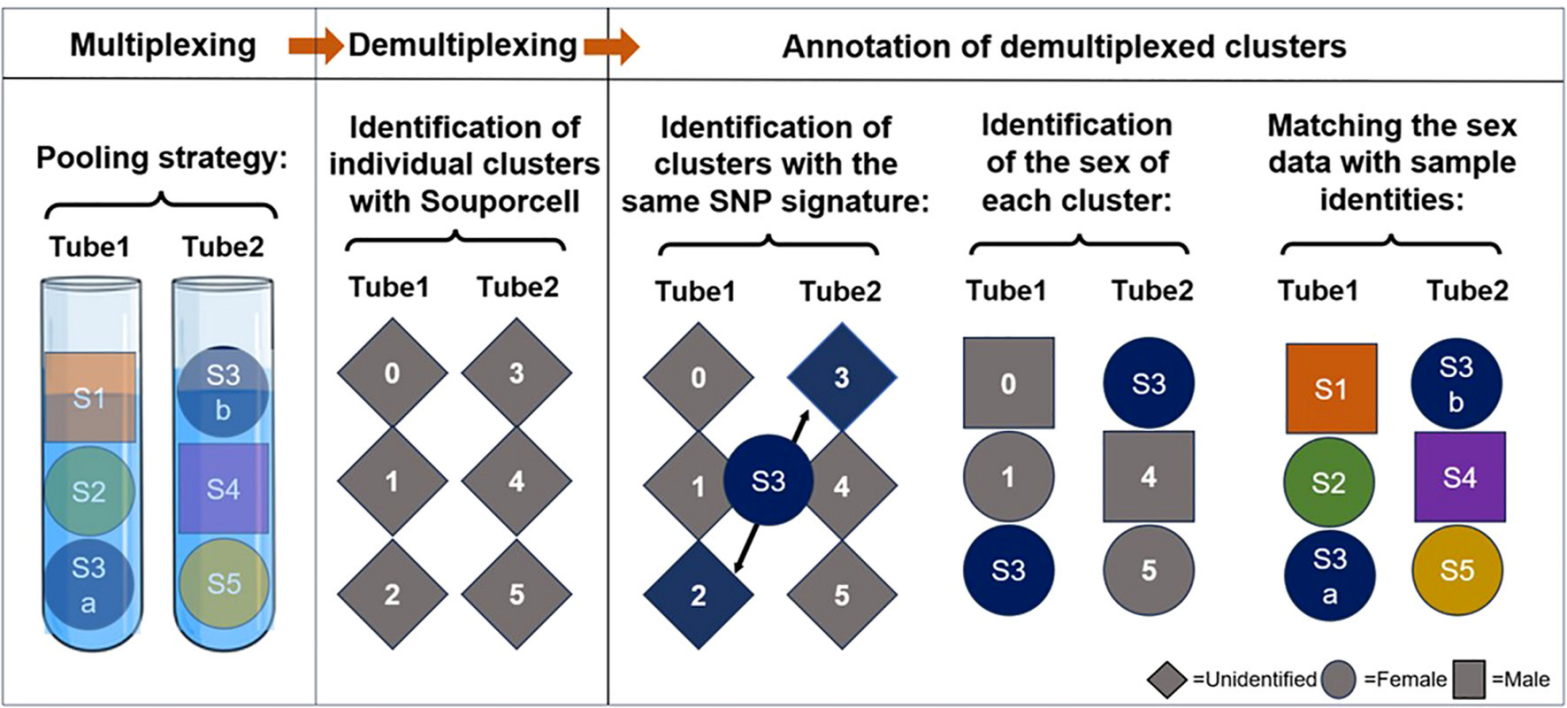
Schematic representation of our label-free triplet pooling strategy. First, we segregate paired PBMC (S3a) and dissociated skin samples (S3b) from a selected individual into two distinct tubes. Additionally, two genetically unrelated samples, each from subjects of different sexes (Female is demonstrated as a circle and Male as a square), are introduced to each tube, resulting in three samples per tube. Following scRNA-seq and sample demultiplexing using souporcell, we first identify the clusters exhibiting identical genetic single nucleotide polymorphism (SNP) patterns in dual reactions and then annotate their sample identity. Subsequently, Y chromosome genes are utilized to ascertain the sex information of the remaining two samples in each tube. By using this information the sample identities of the remaining clusters can be determined readily. S, sample. Different colors denote samples from separate subjects.

Our second, more advanced strategy incorporates two sources of genetic information: the common individual SNP fingerprint of paired samples and the donor sex (**Table 3**, **Figure 5**). In this strategy, paired PBMC and dissociated skin samples are again placed separately in dual reactions. Additionally, two genetically unrelated samples are added to each tube, resulting in three samples per tube. The key point in this step is to select samples from subjects of different sexes for each tube. Following scRNAseq analysis and sample demultiplexing with souporcell, matched sample pairs with identical genetic SNP patterns are identified similar to the first strategy. Subsequently, the sex information of the remaining two samples in each tube is determined. Specifically, the percentage of Y chromosome gene expression among the whole transcriptome is used as a proxy for male sex. With this sex information, the identities of the remaining samples can be easily determined. With a carefully designed setup as proposed in **Table 3**, it is feasible to analyze nine pairs of matched PBMC and skin tissues in 6 reactions, instead of 18.

**Table 3:** An example of the label-free triplet pooling strategy that allows for the analysis of nine pairs of matched PBMC and skin tissues in six reactions, instead of 18.

Reaction no:	Tissue:	Subject code:								
		S1	S2	S3	S4	S5	S6	S7	S8	S9
I	Skin			MP1 PBMC						
II	PBMC			MP1 Skin						
III	Skin								MP2 PBMC	
IV	PBMC								MP2 Skin	
V	Skin									
VI	PBMC									

Blue color shows samples from males and pink shows samples from females. MP: matched pair of samples obtained from the same subject. Detailed descriptions of the first two reactions in this table are illustrated in **Figure 5**.

### Bioinformatics analysis and representative results

**Figure 6:**
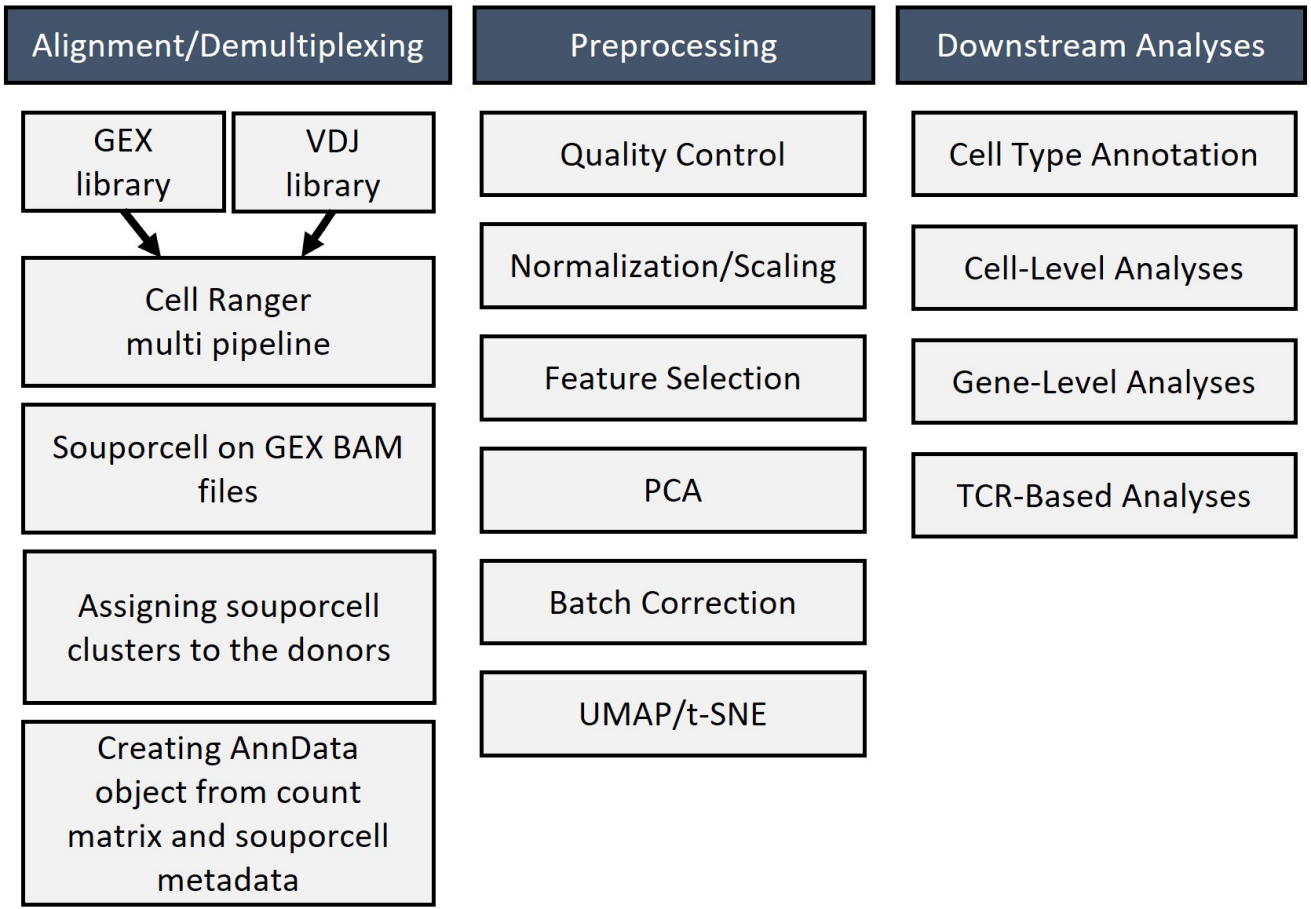
Bioinformatic workflow for analyzing single-cell RNA sequencing results that include alignment, demultiplexing, preprocessing, and downstream analysis of sequencing data.

**Figure 7:**
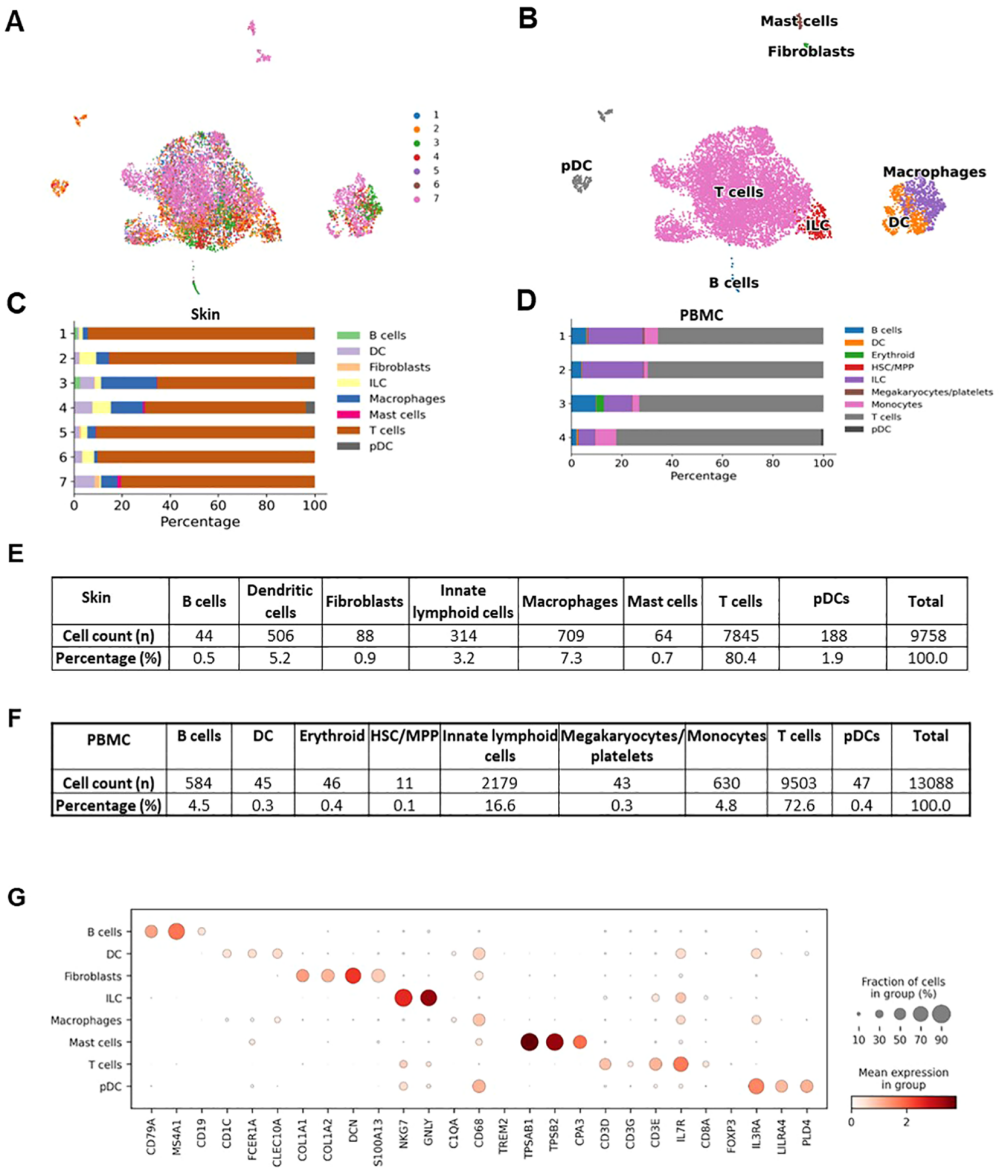
Representative results of the single cell RNAseq analysis of skin samples (n=7) obtained from patients with Behçet’s disease (n=4) and healthy controls (n=3). **(A)** Demultiplexed skin samples are highlighted on UMAP embedding. **(B)** Identified high-hierarchy cell types are visualized. **(C)** Cell type proportion for skin samples from each donor is illustrated with a stacked bar plot. **(D)** Cell type proportions for PBMC samples from 4 Behçet’s disease patients are illustrated with a stacked bar plot. **(E)** The cell counts and proportions of cell types in skin samples are shown. The ratio of fibroblasts is less than 1%, indicating the high purity of the sorted CD45+ cells. **(F)** The cell counts and percentages of cell types in PBMC samples are illustrated. **(G)** Marker genes to characteristic for each cell type are shown as a dot plot.

The individual steps of the bioinformatics analysis pipeline are summarized in **Figure 6**. First, alignment was done with Cell Ranger. Next, demultiplexing was performed with souporcell and the identification of samples was done with the strategy explained above. Then, anndata object was formed and quality control, normalization, feature selection, principal component analysis, and batch correction were done with Scanpy (19). Quality metrics of the scRNAseq data is presented at **Supplementary Figure 4**. UMAP graph showing representative results belonging to the analysis of inflamed skin samples obtained from patients with Behçet’s disease (n=4) and healthy skin (n=3) is presented in **Figure 7A**. Cell type distribution (**Figure 7B**) and percentages (**Figure 7C**), key genetic markers of each cell subset (**Figure 7G**), and cumulative number and percentage of each cell subset (**Figure 7E**) are shown. Also, cell type distribution (**Figure 7D**), cumulative number, and percentage of each cell subset (**Figure 7F**) from 4 PBMC samples of BD patients are illustrated. **Supplementary Figure 5** demonstrates single-cell expression of marker genes across different samples. Comparison of lymphocyte subset frequencies detected by scRNAseq and flow cytometry analyses is shown in **Supplementary Figure 6**. Six samples (3 from Behçet’s disease patients and 3 from healthy controls) illustrated in **Figure 7** were analyzed. No statistically significant differences were observed between the two methods for T cell, T helper cell, cytotoxic T cell, NK cell, Granzyme K+ T cell, and Granzyme B+ T cell percentages (**Supplementary Figure 6A**). Spearman correlation analysis indicated a strong trend towards correlation of T cell percentages measured by these two methods (**Supplementary Figure 6B**).

## Discussion

In this study, we present a novel approach for multiplexing skin and blood samples for single-cell RNA sequencing, significantly reducing costs. Additionally, we provide detailed optimization steps and a step-by-step protocol that enables both scRNAseq and protein-based single-cell analyses from the same tissue sample.

Sample multiplexing is a key strategy to enhance experimental feasibility, mitigate technical batch effects, and significantly reduce the costs associated with scRNAseq. Several demultiplexing methods exist in the literature, such as the use of oligonucleotide-labeled hashtag antibodies to uniquely barcode cells (20). In the scRNAseq protocol for human skin developed by Saluzzo et al., hashtag antibodies were employed for sample de-multiplexing (10). Compared to our label-free de-multiplexing strategy, this method adds additional experimental steps and extra costs due to antibodies, cDNA library preparation, and sequencing and it is limited to multiplexing of two samples.

Another de-multiplexing method is the demuxlet method that leverages single-nucleotide polymorphisms (SNPs) from a genotype reference obtained through whole-genome or exome sequencing, which was previously regarded as the gold-standard de-multiplexing method (21). Recently, the souporcell algorithm was introduced for de-multiplexing samples based on scRNAseq data without requiring a genotype reference or any other label (13). Souporcell generated benchmarking results that surpasses the demuxlet method, showing its huge potential in scRNAseq studies (13). Based on this, we decided to adopt souporcell as the basis of our sample multiplexing strategy.

Although multiplexed samples can be successfully separated using souporcell, the challenge lies in matching these samples to the correct donor. The original souporcell paper proposed a solution by using the same donor-specific sample in multiple reactions. This approach encodes the inclusion or exclusion of each donor as a bit (0 or 1), and samples in the mixtures are then assigned to corresponding donors using this information-theoretic method (13). In our study, we modified this approach by placing matched skin and PBMC samples from the same donor into different reactions. This provided a canonical way to identify the donors of origin. To further enhance this strategy, we incorporated genetic sex information as a second layer of encoding. This two-step approach allowed for successful demultiplexing and donor identification, reducing the number of required reactions and experimental costs by two-thirds.

Both protein-based methods, such as flow cytometry and CyTOF, and RNA-based single-cell studies conducted on lesional skin are surprisingly scarce. One of the main reasons for this is the challenge associated with skin dissociation compared to other tissues. The skin’s dense collagenous structure necessitates more rigorous enzymatic and mechanical processing to obtain a viable cell suspension, which can sometimes compromise cell viability, degrade epitopes, and decrease RNA quality. The recent introduction of automated tissue dissociator systems and skin dissociation kits has provided a standardized and reliable way to perform this step. However, these kits offer a general protocol that needs to be optimized based on the intended experimental methods, such as cell culturing, or ex vivo single-cell RNA or protein analysis from fresh or frozen cells. While recent protocol papers have detailed the use of these kits to prepare skin cell suspensions for scRNAseq analysis of healthy human and pig skin (10, 11), our study expands on these methodologies by optimizing the protocol for inflamed skin. Additionally, we introduce a method suitable for simultaneous flow cytometry analysis from the same tissue, which can also be adapted to other protein-based analyses such as CyTOF and CITE-seq.

The amount of tissue required for analysis is an important consideration. Our findings align with prior research indicating that a 6 mm punch biopsy is optimal for obtaining sufficient skin cells from healthy skin for subsequent scRNAseq analysis (10, 11). Previously, Saluzzo et al. showed that a 6 mm punch biopsy specimen gives a yield of 1-2 × 10^5^ skin cells after incubating with enzyme P for 3 hours. In our study, we found that 3 hours of enzymatic digestion without enzyme P yielded an average of 1.125 × 10^5^ cells, in line with Saluzzo et al. We recommend the usage of two pieces of 6 mm biopsy from healthy skin if both scRNAseq and flow cytometry analysis are planned. For inflamed skin samples, a 4 mm punch biopsy specimen yielded an average of 4.6 × 10^5^ cells, significantly higher than healthy skin, and sufficient for both flow cytometry and scRNAseq analysis. It should be noted that in this study we have only tested Behçet’s Disease skin lesions which are acute inflammatory lesions by their nature. There is a possibility that in more chronic lesions such as psoriasis, systemic sclerosis etc., the degree of inflammation and the number of inflammatory cells may be lower. Therefore, we recommend prior determination of the size of required skin specimen for other lesion types in future studies.

Another important consideration for single-cell studies is whether to analyze cells freshly or after cryopreservation. In a recent study, scRNAseq was performed on freshly isolated cells from 4 mm punch biopsies obtained from patients with atopic dermatitis and psoriasis (14). While fresh analysis of skin samples may seem ideal, it is challenging to coordinate, as all experimental steps—beginning with the collection of biopsy specimens—must be conducted in parallel across multiple patients. In our study, we opted to use cryopreserved cells, which offered several advantages, such as greater flexibility in experimental design, simplified sample multiplexing, and reduced technical batch effects, ultimately lowering scRNAseq costs. Importantly, our assessment of CD45+ lymphocyte viability before and after cryopreservation showed no significant differences in live cell count compared to freshly isolated cells. This finding aligns with previous studies that evaluated the effects of cryopreservation using a 90% FBS + 10% DMSO solution on pig skin cells (11), where cryopreservation did not significantly affect cell viability, aggregation, or gene expression profiles, as demonstrated by scRNAseq analysis.

Custom protocols for skin dissociation have also been used previously. Burja et al. developed a method for skin tissue dissociation for scRNAseq analysis using dispase II, collagenase IV, and trypsin on 4 mm punch biopsies (5). This method yielded a total of 24,053 skin cells per sample from fresh healthy skin and 18,535 cells per sample from skin explants obtained from systemic sclerosis patients cultured for 24 hours. A comparison between cultured skin explants and freshly dissociated samples revealed no significant differences in scRNAseq quality metrics. While the authors observed some alterations in the expression of marker genes in fibroblasts, no such changes were detected in immune cells between freshly dissociated and explant-cultured samples. These findings indicate that the explant method can be an alternative approach for studying the composition of skin immune cells.

The successful generation of GEMs is a key step in scRNAseq using the 10X Chromium system and relies on the presence of high-quality cells in the right concentration. It is important to note that the quality requirements for scRNAseq analysis are higher than those for flow cytometry analysis, and obtaining high-quality cells within the recommended concentration range after fluorescence-activated cell sorting can be challenging. Based on our experience, minimizing the time between cell sorting and GEM generation is essential, a point also noted by Saluzzo et al. (10). In our experimental design, we pooled 2-3 samples for each reaction and conducted two reactions simultaneously using two cell sorters to reduce post-sorting handling time. Careful preparation is crucial to ensure that all necessary materials for GEM generation are ready immediately after cell sorting. Often, the concentration of cells after sorting falls below the recommended range. In such cases, Saluzzo et al. suggest using a volume reduction device (10); however, this equipment may not be available in all laboratories. To address this limitation, we recommend centrifuging the cells at 850 g for 5 minutes after sorting, then reconstituting the cell pellet at a higher concentration than initially desired before recounting the cells, as some may be lost during centrifugation and supernatant removal.

Enzyme P was used in previous tissue dissociation protocols for scRNAseq to increase the cellular yield. However, we do not recommend using enzyme P when protein-based analysis such as flow cytometry or CITE-Seq is planned as this enzyme cleaves cell surface epitopes, including CD8, CD56, and CD69. Of note, CD45 is not significantly affected by enzyme P, therefore cell sorting of live CD45+ leukocytes can still be performed successfully even if this enzyme is used during dissociation.

The duration of enzymatic incubation is an important consideration for single cell studies. We tested the effect of various enzymatic incubation periods on major lymphocyte markers and found that 1-hour, 3-hour, and 16-hour incubations produced similar results. However, we observed that prolonged incubation resulted in a reduction in the percentage of granzyme B-expressing CD8+ cytotoxic T cells, as well as a decrease in CD4 expression on the surface of T cells. Since granzyme B is typically expressed by effector cytotoxic lymphocytes, we concluded that a 3-hour incubation provides an optimal balance between cellular yield and minimizing damage to effector cells. We recommend refraining from longer incubation durations for single cell studies whenever possible.

Recently, Polakova et al. (12) published a protocol for rapid flow cytometry analysis using 4-mm punch biopsies and collagenase IV and DNase I. In this comprehensive study, the authors compared different incubation times of enzymatic digestion, and compared their tissue dissociation method with the whole skin dissociation kit, which included usage of enzyme P. Using their protocol 12,000 CD45+ cells could be obtained from a 4 mm biopsy after 30 minutes of enzymatic incubation. The percentage of major immune cell types (T cells, B cells, NK cells, and ILCs) were similar between both skin dissociation techniques. Moreover, the authors report that 30 minutes of tissue dissociation can be more reliable for the staining of chemokine receptors compared to longer incubation periods.

In conclusion, in this method paper, we describe a novel cost-effective sample multiplexing approach for scRNAseq studies that can be used with both healthy and inflamed skin and can be combined with single-cell protein analysis from the same tissue. The step-by-step protocol and critical optimization steps reported in this paper can be used to design customized single-cell omics experiments by using skin and other solid tissues.

## Data Availability

The original contributions presented in the study are publicly available. This data can be found here: https://figshare.com/articles/dataset/Skin_scRNA-seq_Data/24079362.
