# Supplementary material for: A cost-effective protocol for single-cell RNA sequencing of human skin
Source: Front Immunol. 2024 Oct 30;15:1393017. doi: 10.3389/fimmu.2024.1393017 (PMC11557338; doi:10.3389/fimmu.2024.1393017)
Supplement: Supplementary file 1 [file DataSheet1.docx]

***Supplementary Material***

**Supplementary table 1.** Antibody list used in this study.

| **Target antigen** | **Fluorochrome** | **Clone** | **Brand** |
| --- | --- | --- | --- |
| **CD3** | PE/Cyanine5 | HIT3a | Biolegend |
| **CD4** | PE-Dazzle 594 | OKT4 | Biolegend |
| **CD8** | Alexa Fluor 700 | SK1 | Biolegend |
| **CD14** | APC/Cyanine7 | HCD14 | Biolegend |
| **CD19** | APC/Cyanine7 | HIB19 | Biolegend |
| **CD20** | Brilliant violet 421 | 2H7 | Biolegend |
| **CD45** | Brilliant violet 605 | HI30 | Biolegend |
| **CD45** | PE/Cyanine5 | HI30 | Biolegend |
| **CD56** | APC | 5.1H11 | Biolegend |
| **CD69** | Brilliant Violet 711 | FN50 | Biolegend |
| **Granzyme B** | PE/Cyanine7 | QA16A02 | Biolegend |
| **Granzyme K** | FITC | GM26E7 | Biolegend |
| **Zombie NIR** |  |  | Biolegend |

**Supplementary table 2.** The markers used to identify the cell populations of interest.

| **Cell type** | **Markers** |
| --- | --- |
| NK cells | CD45+CD56+CD3-CD14-CD19- |
| T cells | CD45+CD3+CD14-CD19- |
| Double Negative T cells | CD45+CD3+CD4-CD8- CD14-CD19- |
| T helper lymphocytes | CD45+CD3+ CD4+CD14-CD19- |
| Cytotoxic T lymphocytes | CD45+CD3+ CD8+CD14-CD19- |
| CD69+ T cell (Resident memory T cell) | CD45+CD3+CD69+CD14-CD19- |


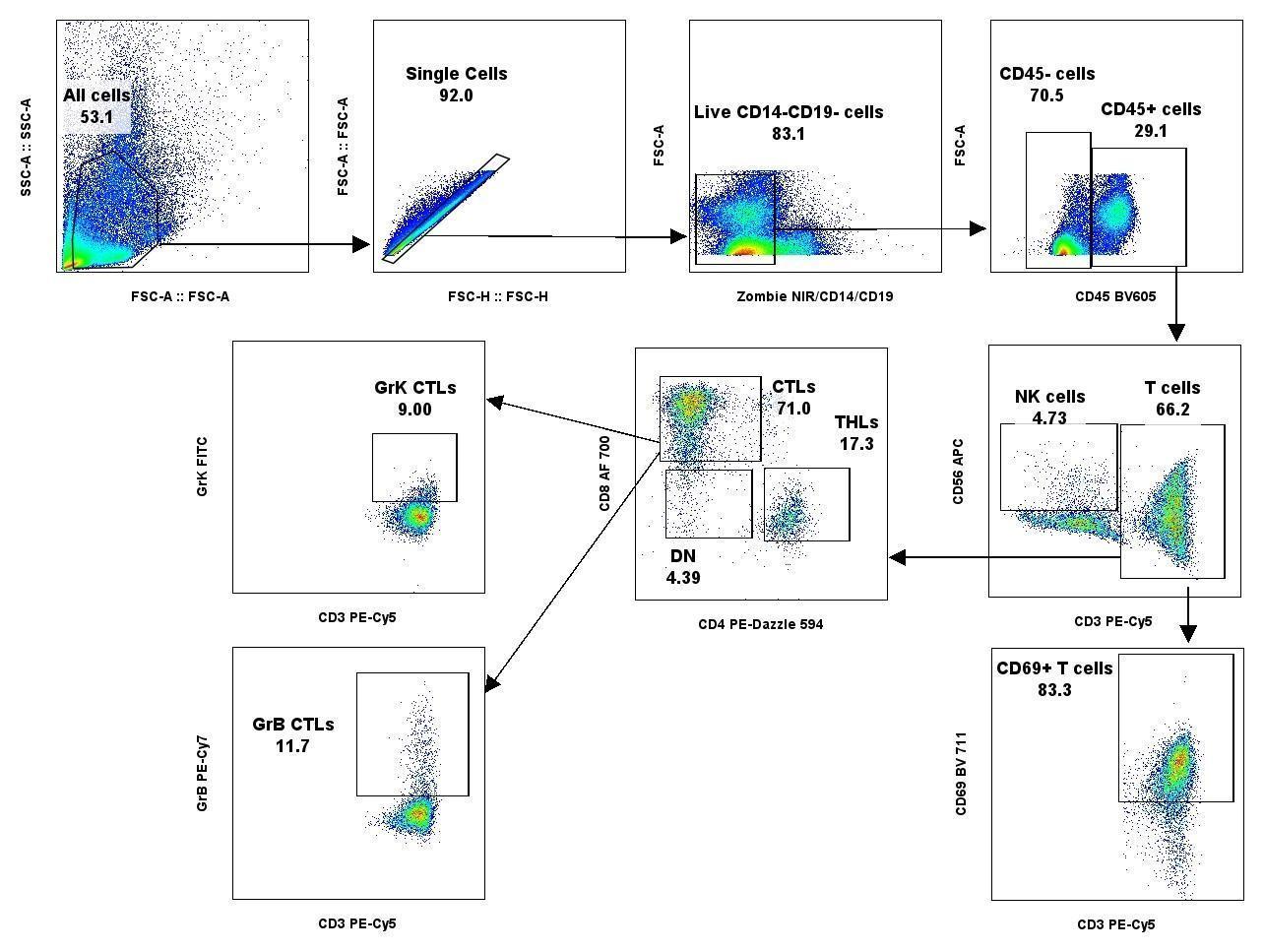


**Supplementary Figure 1.** Representative gating strategy of the flow cytometry analysis. The panel used here was designed to study T cells and NK cells, therefore Zombie NIR, CD14 and CD19 were present together in the APC-Cy7 dump channel. All cells are initially gated via forward scatter (FSC) versus side scatter (SSC). Next, doublets were excluded using FSC-A versus FSC-H parameters. The Zombi NIR-positive dead cells, CD19+ B cells, and CD14+ monocytes that are in the dump channel were excluded. Then, CD45+ leukocytes were gated. After that, CD3+ T cells, and CD56+ NK cells were gated. From T cell population, distinct subtypes including CD69+ T cells (TRMs), CD8+ cytotoxic T cells (CTLs) and CD4+ T helper cells (THLs) were gated. Finally, from the CTLs population, Granzyme K+ and Granzyme B+ cells were gated.


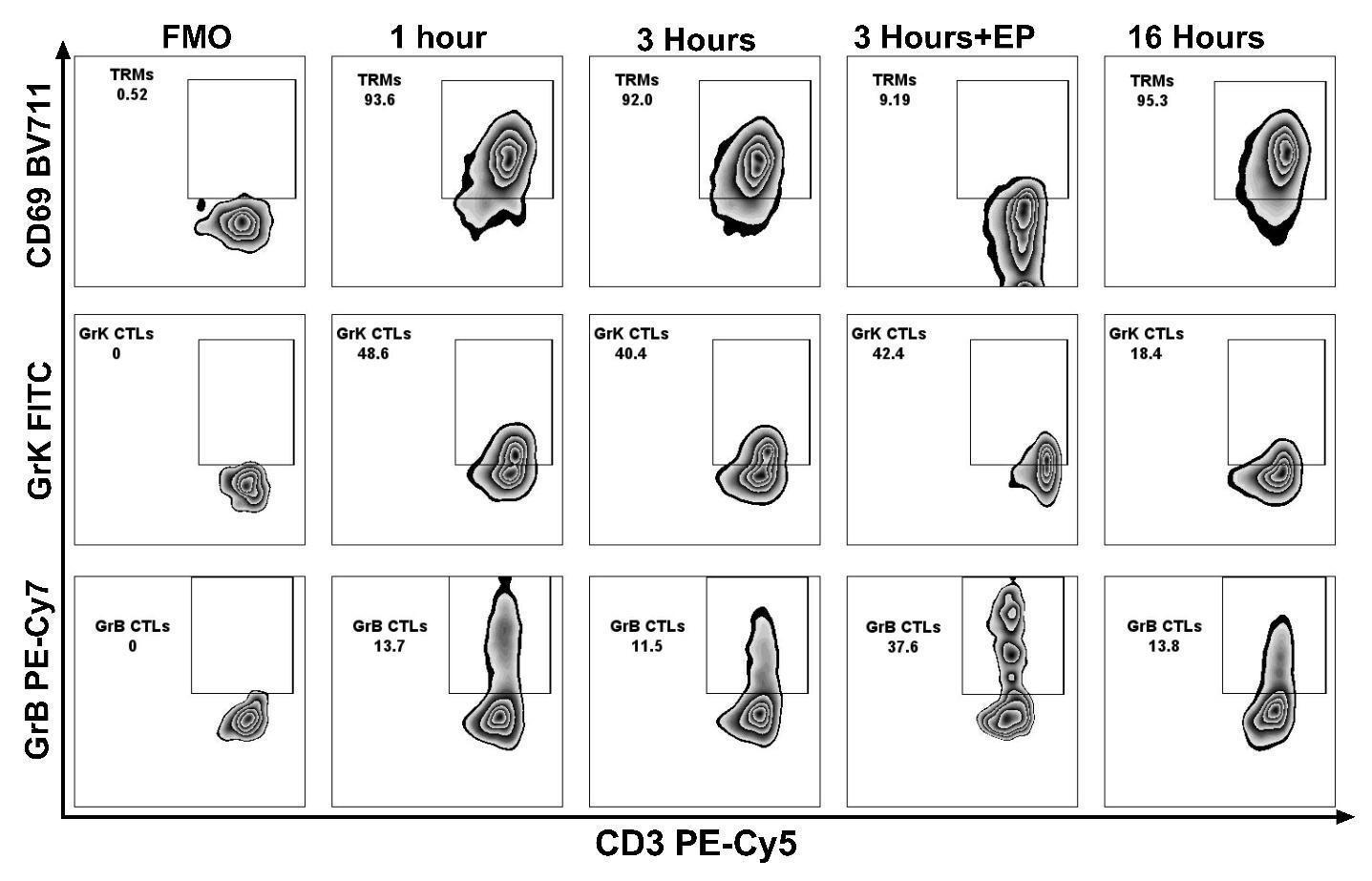


**Supplementary Figure 2.** Fluorescence minus one (FMO) controls for CD69, granzyme K, and granzyme B antibodies.


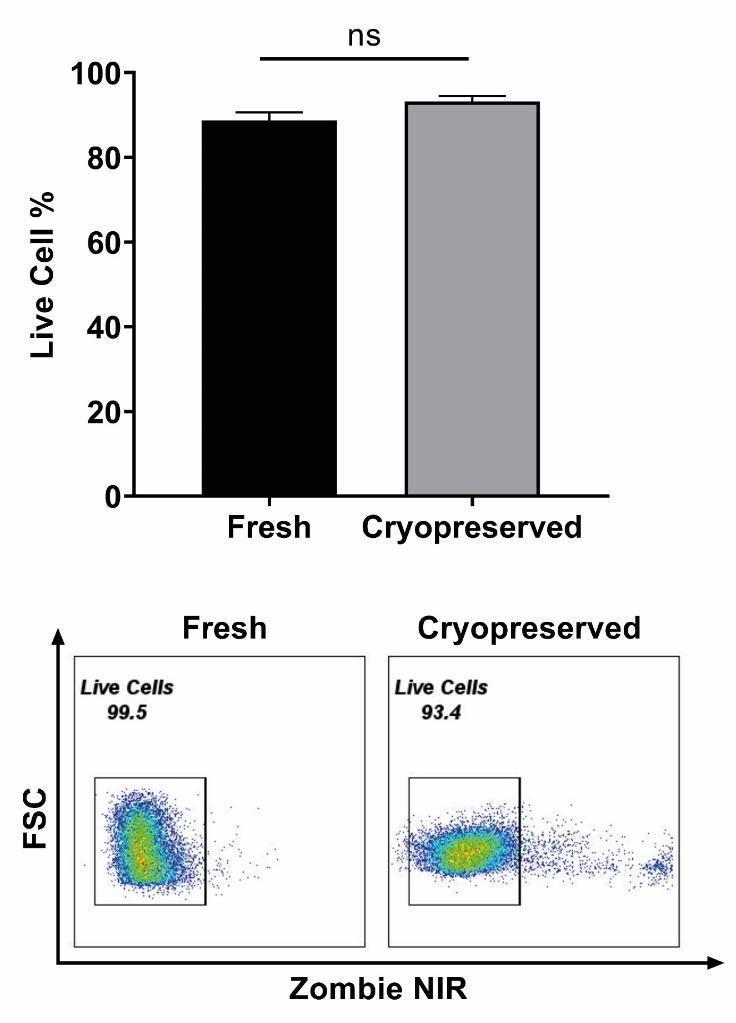


**Supplementary Figure 3.** Freezing and thawing did not make a statistically significant effect on the percentage of live CD45+CD14-CD19- leukocytes, as compared to using freshly isolated cells. Statistical analysis was done with Wilcoxon matched-pairs signed rank test.


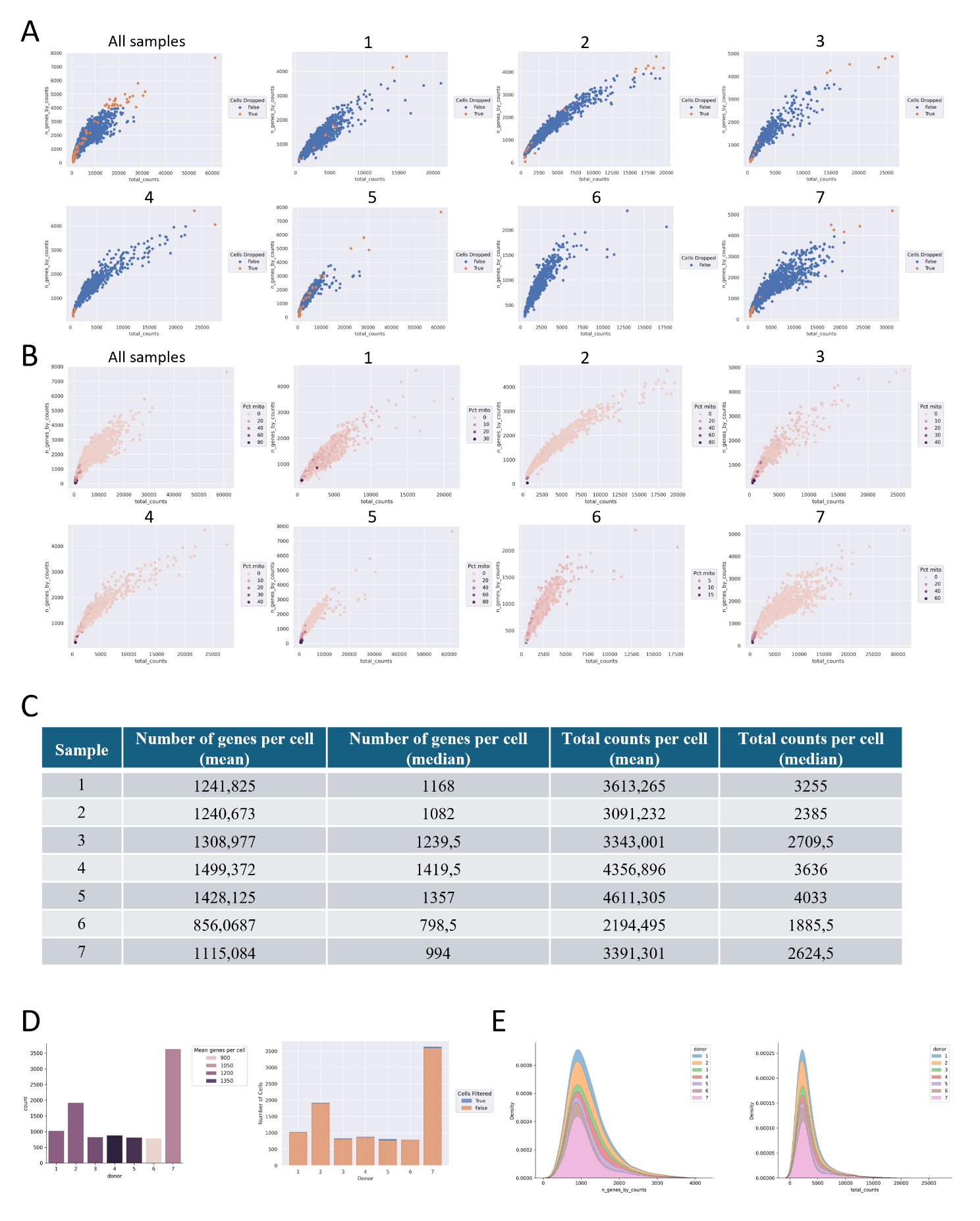


**Supplementary figure 4.** Quality metrics of the scRNAseq data of the seven samples are presented in Figure 7. Scatter plots displaying the total counts (library size) against the number of detected genes per cell. Dot colors indicate whether the cell is retained or dropped. Cells are filtered if marked as doublets (detected by Souporcell or Scrublet) and based on common quality control metrics such as the number of detected genes per cell, total library size, and the percentage of mitochondrial genes. The upper left figure presents data of all samples, while the other plots correspond to samples 1 to 7, from left to right, respectively (A). Scatter plots displaying the total counts (library size) against the number of detected genes per cell. Dot colors represent the percentage of mitochondrial gene expression (B). Table shows the mean and median values for the number of detected genes and total counts per cell (C). Bar plot illustrating cell counts for each sample, with color coding indicating the mean number of detected genes per cell (left). The same bar plot is also color-coded to show whether cells are filtered or not (right) during preprocessing (see Methods) (D). Density plots showing the distribution of number of genes per cell (left) and total counts (right) (E).


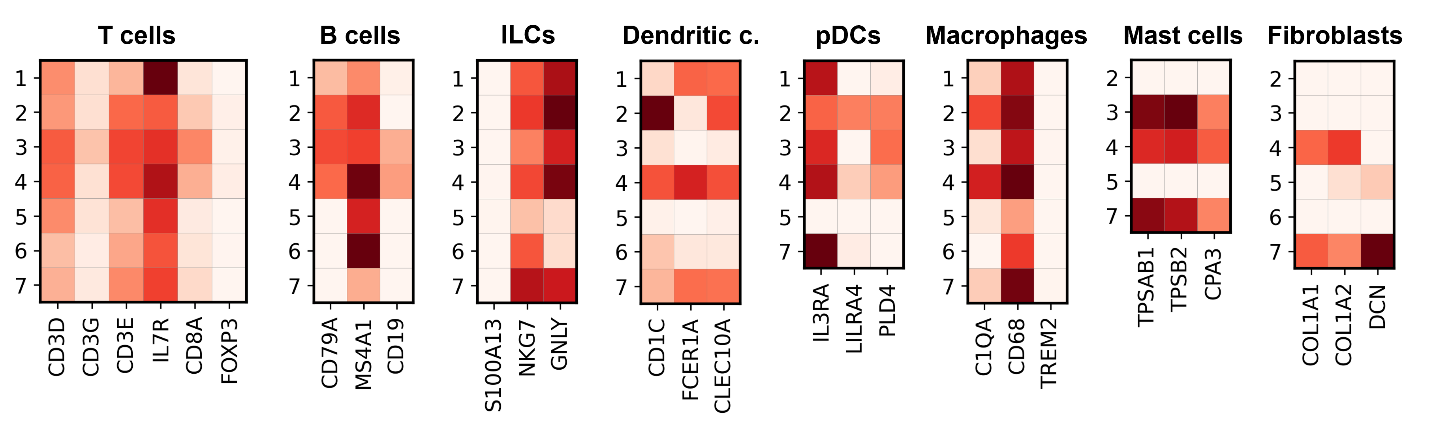


**Supplementary figure 5.** Single-cell expression of marker genes across different samples.


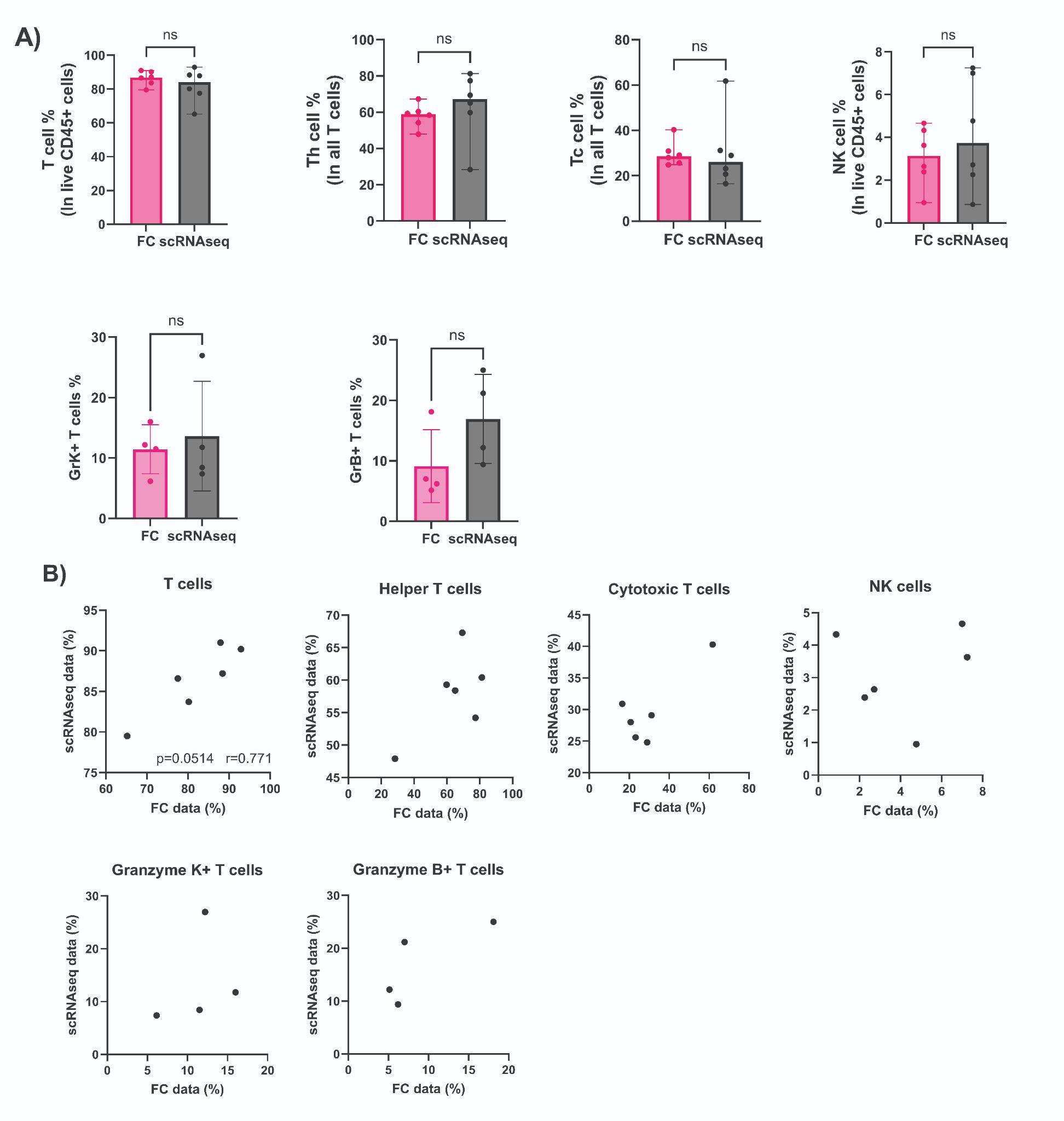


**Supplementary figure 6.** Comparison of lymphocyte subset frequencies detected by scRNAseq analysis with flow cytometry analysis. Six samples (3 Behçet's disease, 3 healthy controls) that were shown in Figure 8 were analyzed. There is no statistically significant difference between two methods for T cell, T helper cell, cytotoxic T cell, NK cell, Granzyme K+ T cell, and Granzyme B+ T cell percentages (A). Spearman correlation analysis showed a trend for the correlation of T cell percentages measured by these two methods (B). Th= T helper cell; Tc= Cytotoxic T cell; FC= Flow cytometry.
