# Supplementary material for: A cost-effective protocol for single-cell RNA sequencing of human skin
Source: Front Immunol. 2024 Oct 30;15:1393017. doi: 10.3389/fimmu.2024.1393017 (PMC11557338; doi:10.3389/fimmu.2024.1393017)
Supplement: Supplementary file 2 [file DataSheet2.docx]

**Protocol for simultaneous FACS and scRNAseq analysis of inflamed and healthy skin samples**

**1. Acquisition of skin samples and preparing the tissue for dissociation:**

In this step, we outline the pre-processing procedures required to prepare fresh skin samples for subsequent tissue digestion.

**Timing: 20 minutes**

- For healthy skin: We recommend using surgically excised skin tissue and obtaining two 6mm punch biopsy specimens *ex vivo* from that excised tissue for subsequent analysis.

- For inflamed skin: A 4mm punch biopsy specimen provides enough cells for both analyses in most cases. However, 6 mm punch biopsy can be preferred whenever possible to make sure that enough cells can be obtained.

**Note:** One 4 mm skin biopsy from BD lesions, results in cell numbers adequate for both flow cytometry and scRNA-seq analysis. However, for healthy skin samples, 2 pieces of 6 mm biopsy are needed for both analyses.

- Skin samples are washed thoroughly with phosphate-buffered saline (PBS) and subcutaneous fat is removed carefully with a scalpel. Then the sample was cut into small pieces with a scalpel.

**Key point:** Be careful not to damage the dermis.

**2. Skin dissociation:**

In this step, we dissociate skin tissue pieces into a single-cell suspension using an enzymatic and mechanical tissue dissociation protocol. This process employs a whole skin dissociation kit and the gentleMACS Octo Dissociator with heaters (Miltenyi Biotec, Germany).

**Timing: 3.5 hours**

- Dissociate the skin tissues enzymatically and mechanically using the Whole Skin Dissociation Kit and gentleMACS Octo Dissociator with heaters (Miltenyi Biotec, Germany) according to the protocol provided by the manufacturer. Briefly, one 4mm specimen of inflamed skin is cut into small pieces by a scalpel and put inside a C tube. For healthy samples, two 6mm specimens are minced into small pieces by a scalpel and placed into a C tube. Then, enzyme A and enzyme D are added into the C tube and left for incubation for 3 hours in a 37 °C water bath. Next, 500 µm of cold DMEM plus 10% fetal bovine serum (FBS) (10500064, Gibco, USA) is added, and mechanical dissociation is performed with gentleMACS Octo Dissociator with the program of "h_skin_01".

**3. Preparation of cell suspension:**

In this step, potential cell debris and aggregates are removed by filtering. The cell yield is then determined. Cell suspension is divided into two portions: one for flow cytometry and the other cryopreserved for future single-cell RNA sequencing experiments.

**Timing: 30 minutes**

- After skin dissociation, centrifuge the samples in the C tube (350 g for 1 minute at 4 °C).

- Filter the collected cell suspension into a 15 mL tube using the 70µm pre-separation filter. Wash the remaining cells on the filter with 4 mL of cold culture medium.

- Centrifuge the cell suspension at 350 g for 10 minutes. Resuspend the cell pellet in 1 mL of PBS and count with a hemacytometer.

**Note:** 10^5^ cells are used freshly for flow cytometry analysis (Step 4). The rest of the cells are cryopreserved for future scRNAseq analysis.

**4. Cell staining for the intracellular and extracellular antigens and flow cytometry analysis:**

In this step, cells are stained with surface and intracellular antibodies, and then analyzed using a flow cytometry device.

**Timing: 2.5 hours**

- Wash freshly isolated skin cells with PBS (Add 1 ml PBS, centrifuge at 500 g for 5 minutes, then remove the supernatant).

- Incubate with Zombie NIR fixable viability dye (423106, Biolegend, USA) for 10 minutes on ice in the dark.

- Add 2 mL of FACS buffer (PBS+ 1% BSA) to the tubes. Centrifugate at 500 g for 5 minutes.

- Remove the supernatant, add the cell surface antibody cocktail, and incubate the cells for 20 minutes on ice in the dark.

- Wash the cells with FACS buffer. Then, incubate with 500 µL of Fixation Buffer (420801, BioLegend, USA) for 20 minutes at room temperature, and then centrifuge at 500 g for 5 minutes.

- Wash the cells with 1 mL Intracellular Staining Permeabilization Wash Buffer (421002, BioLegend, USA) and incubate with the desired intracellular antibody cocktail for 20 minutes at room temperature in the dark.

-Wash the cells with 1 mL Intracellular Staining Permeabilization Wash Buffer and resuspend the pellet in 500 μL of FACS buffer.

- Analyze the stained cells with a flow cytometer.

**5. Design of the sample multiplexing strategy (Optional step):**

**-** If you are planning to multiplex 2-4 samples per each reaction of the Chromium Next GEM Single Cell 5' Reagent Kit V2 (Dual Index, 10X Genomics, USA), you can use either of two options:

i) You can use barcodes to label each sample as recommended in the protocol by Saluzzo et al. Please note that an additional library for the barcodes must be prepared in addition to transcriptomics and TCR/BCR libraries. If this step is not performed, it will not be possible to demultiplex the samples.

ii) The label-free sample multiplexing strategy proposed in the current study can be used. In that case, paired blood and skin samples and samples belonging to different sexes are used for multiplexing two or more samples. Please refer to Figure 6, Table 1, and Table 2 for more information. In this method, demultiplexing is performed by the souporcell algorithm after the library construction and sequencing steps.

**6. Sample preparation for scRNAseq analysis:**

In this step, cryopreserved cells are thawed, CD45+ live cells are sorted, and then prepared for Gel Bead-in-Emulsion (GEM) generation in subsequent steps.

**Timing: 75 minutes**

- Thaw the cryopreserved cell suspensions in a 37^o^C water bath. Add 5 mL of RPMI, centrifuge at 500 g for 5 minutes.

-Resuspend the pellets in PBS and centrifuge again at 500 g for 5 minutes.

- Incubate the cells with Zombie NIR in PBS for 10 minutes on ice in the dark.

- Wash the cells with FACS buffer.

- Incubate the cells with anti-human CD45 PE-Cy5 for 20 minutes.

- Wash the cells with FACS buffer.

- Resuspend the cells in PBS and sort the CD45+ live cells with a cell sorter.

**Note:** During sorting, we recommend using a 100 µm nozzle with a constant pressure of 20 psi. Maintain the sample chamber at 4°C. Keep the sorting speed low (1500 events/seconds) to ensure high viability and purity.

**Key point:** If multiplexing of 2-4 samples is planned, we recommend using two different cell sorters simultaneously, whenever possible. This will significantly reduce the time required for sorting all samples and will result in higher quality samples for scRNAseq. If sorting session lasts longer than 1 hour, this may result in lower quality samples. We do not recommend processing more than 4 samples at one session.

**6. Library preparation and sequencing:**

For library preparation, the steps provided in the manufacturer’s manual should be followed. We recommend using a core facility whenever possible. If you will perform the steps yourself, we recommend preparing by using the video tutorials in the 10X Genomics website. A summary of the experimental steps is provided below.

- Centrifuge the sorted cells at 850 g for 5 minutes at 4 °C and count with a hemacytometer or automatic cell counter.

- Reconstitute the cells in the required volume of PBS to reach the optimum cell concentration of 700 to 1200 cells per microliter, as recommended in the manufacturer’s protocol for the Chromium Next GEM Single Cell 5' Reagent Kit V2 (Dual Index, 10X Genomics, USA).

- Generate GEMs using the Chromium Controller system and the Chromium Next GEM Single Cell 5’ Reagent Kits V2 (Dual Index, 10X Genomics, USA).

- Produce barcoded cDNAs from poly-adenylated mRNAs and purify them using magnetic beads.

- Perform enzymatic fragmentation, size selection, and PCR amplification for library construction.

- Check the DNA quality and quantify using the Agilent Bioanalyzer or Agilent TapeStation.

- Perform paired-end sequencing. A minimum reading depth of 20,000 reads per cell is recommended.

**7. Demultiplexing of samples:**

- Align raw FASTQ files to the GRCh38 reference genome using the Cell Ranger (v.7.1.0) multi-pipeline.

- Perform the first layer of demultiplexing of pooled samples using the souporcell algorithm. The BAM file generated by Cell Ranger is utilized in the souporcell pipeline.

- Determine the identity of each cluster based on the presence of matched skin and PBMC pairs, which share the same genotype in dual reactions. Skin and PBMC samples from the same donor are identified using the “shared_samples.py” module of souporcell.

- The second layer of demultiplexing based on the donor’s sex for each souporcell cluster is determined by analyzing a subset of Y chromosome genes including ZFY, RPS4Y1, EIF1AY, KDM5D, NLGN4Y, TMSB4Y, UTY, DDX3Y, and USP9Y.

**8. Analysis of the single-cell RNAseq data:**

- Process the count matrices using Scanpy. Filtered out the cells with less than 200 genes or more than 4000 genes, and genes expressed in fewer than 3 cells.

- Eliminate the cross-genotype doublets detected by souporcell.

- Use Scrublet to identify doublets based on expression profiles and eliminate.

- Normalize the data to 10,000, and apply log(x+1) transformation.

- Perform feature selection using the “sc.pp.highly_variable_genes” function. PCA is computed on the scaled expression matrix of the highly variable genes.

- BBKNN’s ridge regression function is utilized to eliminate technical confounders such as donor-specific variation and count depth, while preserving biological variation such as cell types and disease effects.

- Integrate different pools using the Harmony algorithm.

- Compute a neighborhood graph and UMAP embedding based on the harmony-corrected principal components.

- Cell type annotation can be performed by CellTypist using pre-trained built-in models such as “Immune_All_High.pkl” and “Immune_All_Low.pkl”. Majority voting classifier is enabled during cell type prediction to increase annotation accuracy.
